# Association of Genetic Polymorphisms with Abdominal Aortic Aneurysm in the Processes of Apoptosis, Inflammation, and Cholesterol Metabolism

**DOI:** 10.3390/medicina59101844

**Published:** 2023-10-17

**Authors:** Nyityasmono Tri Nugroho, Monika Herten, Giovanni F. Torsello, Nani Osada, Elena Marchiori, Sonja Sielker, Giovanni B. Torsello

**Affiliations:** 1 Department of Vascular and Endovascular Surgery, University Hospital Münster, 48149 Münster, Germany; 2 Vascular and Endovascular Division, Department of Surgery, Cipto Mangunkusumo National Hospital, Faculty of Medicine, University of Indonesia, Jakarta 10430, Indonesia; 3 Department of Trauma, Hand and Reconstructive Surgery, University Hospital Duisburg-Essen, 45147 Essen, Germany; 4 Institute of Radiology, University of Göttingen, 37075 Göttingen, Germany; 5 Research Unit Vascular Biology of Oral Structures (VABOS), Department of Cranio-Maxillofacial Surgery, University Hospital Münster, 48149 Münster, Germany; 6 Institute for Vascular Research, St. Franziskus Hospital, 48145 Münster, Germany; giovanni.b.torsello@gmail.com

**Keywords:** abdominal aortic aneurysm (AAA), single nucleotide polymorphism (SNP), inflammation, genetic analysis, aneurysm screening

## Abstract

*Background and Objectives*: This study aims to identify the minor allele of the single nucleotide polymorphisms (SNPs) *DAB2IP* rs7025486, *IL6R* rs2228145, *CDKN2BAS* rs10757278, *LPA* rs3798220, *LRP1* rs1466535, and *SORT1* rs599839 in order to assess the risk of abdominal aortic aneurysm (AAA) formation and define the linkage among these SNPs. *Materials and Methods*: A case-control study with AAA patients (AAA group) and non-AAA controls (control group) was carried out in a study population. DNA was isolated from whole blood samples; the SNPs were amplified using PCR and sequenced. *Results:* In the AAA group of 148 patients, 87.2% of the patients were male, 64.2% had a history of smoking, and 18.2% had relatives with AAA. The mean ± SD of age, BMI, and aneurysmal diameter in the AAA group were 74.8 ± 8.3 years, 27.6 ± 4.6 kg/m^2^, and 56.2 ± 11.8 mm, respectively. In comparison with 50 non-AAA patients, there was a significantly elevated presence of the SNPs *DAB2IP* rs7025486[A], *CDKN2BAS* rs10757278[G], and *SORT1* rs599839[G] in the AAA group (*p*-values 0.040, 0.024, 0.035, respectively), while *LPA* rs3798220[C] was significantly higher in the control group (*p* = 0.049). A haplotype investigation showed that the SNPs *DAB2IP*, *CDKN2BAS*, and *IL6R* rs2228145[C] were significantly elevated in the AAA group (*p* = 0.037, 0.037, and 0.046) with minor allele frequencies (MAF) of 25.5%, 10.6%, and 15.4%, respectively. Only *DAB2IP* and *CDKN2BAS* showed significantly higher occurrences of a mutation (*p* = 0.028 and 0.047). Except for *LPA*, all SNPs were associated with a large aortic diameter in AAA (*p* < 0.001). Linkage disequilibrium detection showed that *LPA* to *DAB2IP*, to *IL6R*, to *CDKN2BAS*, and to *LRP1* rs1466535[T] had *D’* values of 70.9%, 80.4%, 100%, and 100%, respectively. *IL6R* to *LRP1* and to *SORT1* had values for the coefficient of determination (*r^2^*) of 3.9% and 2.2%, respectively. *Conclusions*: In the investigated study population, the SNPs *CDKN2BAS* rs10757278, *LPA* rs3798220, *SORT1* rs599839, *DAB2IP* rs7025486, and *IL6R* rs2228145 were associated with the development of abdominal aortic aneurysms. Individuals with risk factors for atherosclerosis and/or a family history of AAA should be evaluated using genetic analysis.

## 1. Introduction

Abdominal aortic aneurysm (AAA) is a degenerative disease with a prevalence from 3.9 to 7.7% in the USA and Europe [1]. It mainly occurs in the last decades of life and predominantly affects the male gender [2]. A ruptured AAA leads to a high mortality rate of up to 80% [3,4]. The risk of developing AAA increases with hypertension, dyslipidemia, and a history of smoking [5,6]. The impact of the risk factor “smoking” is expressed in a high odds ratio (OR) between smoking and non-smoking AAA patients of 2.3 to 13.72 [6]. Hereditary factors also play a role in the development of AAA with up to 20% of all AAA cases attributable to genetic predisposition [7,8]. The prevalence of familial AAA is from 13 to 25% and is found among siblings [7]. Various investigators have studied the polymorphisms of specific genes and encoded key molecules that are likely to be involved in AAA formation, such as structural proteins of the vessel wall, tissue-degrading enzymes and corresponding tissue inhibitors, immuno-modulatory molecules, and molecules involved in hemodynamic stress [4]. In meta-analyses of genome-wide association studies (GWAS) with distinct ethnicities and population genetic studies, different genes with single nucleotide polymorphisms (SNPs) have been reported to be associated with AAA [3,9,10]. The following six gene sequences and corresponding SNPs *DAB2IP* (rs7025486), *IL6R* (rs2228145), *CDKN2BAS* (rs10757278), *LPA* (rs3798220), *LRP1* (rs1466535), and *SORT1* (rs599839) have not yet been investigated in the study population, for which only a few studies have reported on other SNPs and their association with AAA [11].

The aim of our study is to identify the minor allele of the SNPs to assess the risk of AAA in a study population and define the linkage among these SNPs.

## 2. Materials and Methods

### 2.1. Study Population

This is a single-center case-control study of AAA patients and non-AAA controls in two referral centers for vascular and endovascular surgery between November 2016 and October 2019. All individuals provided their written informed consent to participate in this study. The study was conducted according to the Declaration of Helsinki and passed ethical clearance from the Ethics Commission of the Medical Council (Approval Number 2016-361-f-S).

The inclusion criteria were a confirmed diagnosis of degenerative AAA and patient age ≥18 years, with and without type II diabetes mellitus. The exclusion criteria were aortic dissection, connective tissue disorders, e.g., Ehlers-Danlos syndrome, Loeys-Dietz syndrome, or Marfan syndrome, and HIV/Hepatitis C infection. The control group consisted of patients ≥18 years who were previously not diagnosed with AAA.

Patients and controls were selected consecutively in the ambulatory setting. All relevant clinical information was collected from the patients’ histories and the medical record system.

### 2.2. Genotyping

Whole blood samples (8.5 mL) were collected from the patients and the controls using PAXgene Blood DNA Tubes (PreAnalytiX Qiagen, Hilden, Germany). DNA isolation was performed according to the protocol of the PAXgene Blood DNA Kit (Qiagen). Primers and the PCR protocol are listed in Supplementary Table S1. The PCR products were controlled using gel electrophoresis (Supplementary Figure S1). The detected gene sequences were purified using a PeqGOLD-Microspin Cycle-Pure Kit (VWR International, Darmstadt, Germany). The PCR products were sequenced using the Sanger method performed by GATC (Biotech-AG-Eurofins, Hamburg, Germany). The locations of the polymorphism loci of the SNPs were according to the dbSNP (NCBI). The polymorphism loci were evaluated using SNAPGene Viewer (GSL-Biotech-LLC, Chicago, IL, USA) and the software program Clustal-Omega (EMBL-EBI, Cambridge, UK).

### 2.3. Statistical Analysis

All statistical data and Forest plot diagrams were calculated using SPSS 27 (IBM, New York, USA). The nominal or ordinal parameters were presented as frequencies and percentages, and the numeric parameters were presented as mean ± SD (standard deviation). The categorical parameters were analyzed using the χ^2^ test and Fisher’s Exact test. Steady-state parameters were analyzed using the Mann–Whitney test or Wilcoxon test. A Student’s *t*-test was performed for comparison of the numeric data. Logistic regression univariate analysis was performed to calculate the OR (odds ratio) with 95% CI (confidence interval). The significance level was tested bilaterally with *p*-value < 0.05. Forest plot diagrams were used to summarize the OR of all six SNPs in this study using the lower and upper values of the 95% CI as limits.

Sample size estimation for genetic predisposition was performed using the software Power and Sample Size Calculation (Dept. of Biostatistics, Vanderbilt University, Nashville, TN, USA). To ensure homogeneity in the sex and age structure of the control group, recruitment of the controls was planned on the basis of the sex and age distribution in the AAA group.

The Hardy–Weinberg Equilibrium (HWE) describes a stationary state of the genetic variation (allele frequency) in a normal population from one generation to the next generation in the absence of other evolutionary changes, as opposed to the HWD—Hardy–Weinberg Disequilibrium. Deviations from HWE were tested using Pearson’s χ^2^ test, which evaluated the degree of difference between the observed genotype and allele frequencies and the frequencies that were expected if the HWE assumption held. Statistically significant test results suggest deviation from the HWE assumption.

Linkage Disequilibrium (LD) is the nonrandomized association of alleles at two or more loci. It is expressed as the basic linkage disequilibrium parameter, *D*, which is the difference between the observed and expected haplotype frequencies and is expressed as a percent. To avoid negative *D* in this study, we used *D’* as the result of *D/D_max_*. A metric of LD is *r^2^*, which is equivalent to the Pearson correlation coefficient. *r^2^* is calculated as a quotient of *D^2^* and the product of frequencies and ranges from 0 to 1. TheLD was calculated using HaploView 4.2 (Broad-Institute, Cambridge, USA).

## 3. Results

### 3.1. Patient Characteristics

In total, 153 AAA patients and 54 controls were recruited. The rate of dropout samples was 3.3% (*n* = 5/153) in the AAA patients and 7.4% (n = 4/54) in the control group due to either insufficient DNA yield or detection of an HIV infection after blood analysis. The data of 148 AAA patients and 50 controls were evaluated. Patients with AAA were predominantly male, aged >65 years, and displayed significantly more comorbidities such as arterial hypertension, dyslipidemia, and a history of smoking compared to the controls (*p* < 0.001) (Table 1). Peripheral artery disease (PAD) was diagnosed in 25 AAA patients (16.9%).

A total of 103 AAA patients (69.6%) had a maximum aortic diameter ≥50 mm. The morphology was mostly fusiform (n = 111/148, 75%) and the aneurysms were located infrarenal (n = 95/148, 64.2%) (Supplementary Table S2).

Significant risk factors for a genotype mutation risk of AAA development (minor homozygotes and heterozygotes vs. major homozygotes) were investigated (Table 2). A higher occurrence of the SNP *DAB2IP* rs7025486[A] was detected in AAA patients with arterial hypertension (*p* < 0.001 with OR 3.295, 95% CI [1.704–6.374]) while a significantly higher occurrence of the SNP *LRP1* rs1466535[T] was found in patients with a family history of AAA (*p* = 0.005; OR 3.275, 95% CI [1.390–7.717]) when compared to the control group. Obesity was significantly more often associated with AAA development for the genotype mutation of *SORT1* rs599839[G] (*p* = 0.025, OR 2.419, 95% CI [1.101–5.314]). For *LPA* rs3798220[C] and *IL6R* rs2228145[C], there were no significant differences in the occurrence of genotype mutations between the AAA group and the controls.

### 3.2. Allele Frequencies, Haplotypes, and Mutations of the SNPs

#### 3.2.1. Allele Frequencies

**Figure 1:**
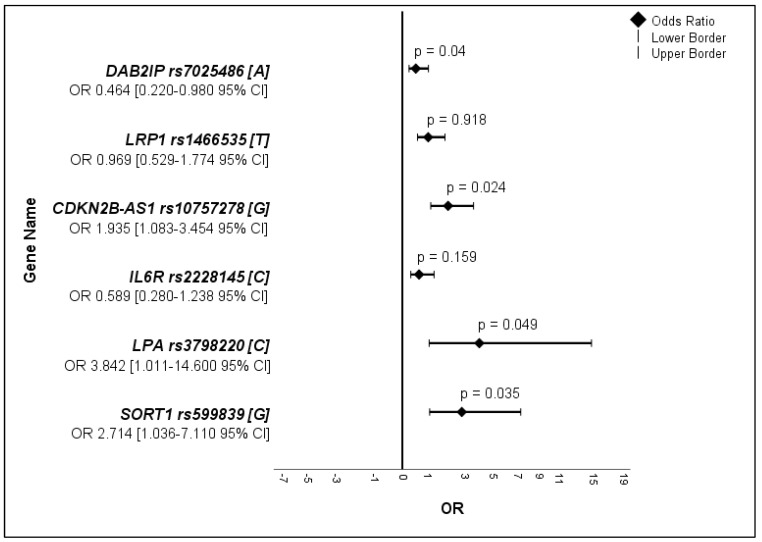
Forest plot of all SNPs.

For the allele frequency analyses, both alleles of the gene were considered, resulting in 296 alleles for the AAA group and 100 alleles for the control group (Table 3, Figure 1).

The minor allele of the SNP *DAB2IP* rs7025486[A] was present in 17.6% (n = 52/296) of the alleles in the AAA group and in 9% (n = 9/100) of those in the control group. The overall OR between the two groups was 0.464, 95% CI [0.220–0.980], *p* = 0.040 with an OR < 1, indicating a decreased occurrence of the SNP *DAB2IP* rs7025486[A] in AAA development (protective exposure). Besides the SNP *DAB2IP* rs7025486[A], the SNPs *CDKN2BAS* rs10757278[G], *LPA* rs3798220[C], and *SORT1* rs599839[G] also displayed significant differences in allele frequencies between the AAA and control groups. Here, the OR > 1 indicated the increased occurrence of these SNPs in AAA. The minor allele of *CDKN2BAS* rs10757278[G] was detected in 25% (n = 74) of male AAA patients and in 3.4% (n = 10) of female patients, with an OR for both sexes of 1.935, 95% CI [1.083–3.454], *p* = 0.024. The minor allele of *LPA* rs3798220[C] was displayed in 1.4% (n = 4) of the male AAA patients and was not detected in female AAA patients, but was detected in 3% (n = 3) of the female controls. The OR for both sexes was 3.842, 95% CI [1.011–14.600] in the AAA group with *p* = 0.049. The SNP *SORT1* rs599839 showed a distribution of the minor allele [G] in 10.8% (n = 32) of the male and in 1.7% (n = 5) of the female AAA patients and was significantly higher than in the control group, *p* = 0.035. The OR for both sexes in the AAA group was 2.714, 95% CI [1.036–7.110]. The allele frequencies of *LRP1* rs1466535 [T] and *IL6R* rs2228145[C] did not show significant differences between the AAA and the control groups for either sex (*p* = 0.918 and 0.159, respectively).

#### 3.2.2. Haplotypes

Regarding zygosity, the similarities or differences between the individuals’ alleles, the set of DNA variations (polymorphisms) adjacent to one another at the same locus that tend to be inherited together (haplotypes), are presented in Table 4. For each SNP, the frequencies for major homozygote, heterozygote, and minor homozygote alleles are listed. In addition, the minor allele frequency (MAF) indicating the percent or fraction of the second most common allele for a given locus in a population is specified. The most frequent minor allele was detected in *DAB2IP* rs7025486[A] with a minor allele frequency (MAF) of 25.5% (*p* = 0.037). In the AAA group, this SNP had a frequency for major homozygote (GG), heterozygote (GA), and minor homozygote (AA) of 70.3% (n = 104), 24.3% (n = 36), and 5.4% (n = 8), respectively, for both sexes. The other two SNPs with significant differences in haplotypes were *CDKN2BAS* rs10757278[G] (*p* = 0.037; MAF = 10.6%) and *IL6R* rs2228145[C] (*p* = 0.046; MAF = 15.4%). The SNPs *LRP1* rs1466535[T], *LPA* rs3798220[C], and *SORT1* rs599839[G] showed no significant differences in haplotypes (*p* = 0.835, 0.146, and 0.203, respectively).

#### 3.2.3. Mutations

The mutation occurrences in each genetic polymorphism revealed a higher occurrence of the SNP *DAB2IP* rs7025486[A] in the AAA group with 29.7% (n = 44) vs. 14% (n = 7) in the control group (*p* = 0.028) (Table 5). Also, the SNP *CDKN2BAS* rs10757278[G] showed a mutation in 43.9% (n = 65) of the AAA patients which was significantly higher than in the control group (28%, n = 14) (*p* = 0.047). For the SNPs *LRP1* rs1466535[T], *IL6R* rs2228145[C], *LPA* rs3798220[C], and *SORT1* rs599839[G], no significant differences in mutation occurrence were detected (*p* = 0.755, 0.073, 0.113, and 0.100, respectively).

In conclusion, the SNPs *DAB2IP* rs7025486[A] and *CDKN2BAS* rs10757278[G] were significantly different in all three investigated parameters: allele frequencies, haplotypes, and mutation occurrences, while no difference was detected for the SNP *LRP* rs1466535[T] (*p* = 0.918, 0.835, 0.755, respectively) (Table 3, Table 4 and Table 5).

Aneurysm sac size (>50 mm versus <50 mm) and morphology of AAA (saccular versus fusiform) revealed significant differences between the parameters in all SNPs, except for LPA rs3798220[C] (*p* = 0.180 and 0.401) (Table 6). With regard to the supra/juxtarenal vs. infrarenal AAA location, significant differences occurred only in the SNPs *IL6R* rs2228145[C], *LPA* rs3798220[C], and *SORT1* rs599839[G] (*p* < 0.001, *p* < 0.001, and *p* = 0.005, respectively).

Regarding the Hardy–Weinberg Equilibrium (HWE), the four SNPs LRP1 rs1466535[T], *CDKN2BAS* rs10757278[G], *IL6R* rs2228145[C], and *SORT1* rs599839[G] were in accordance with the HWE in the control group (*p* = 0.578, 0.119, 0.335, and 0.709, respectively) (Supplementary Table S3). In contrast to these, the SNPs *DAB2IP* rs7025486[A] and *LPA* rs3798220[C] were in accordance with the Hardy–Weinberg Disequilibrium (HWD) in the control group (*p* = 0.006 and 0.009, respectively).

### 3.3. Linkage Disequilibrium

**Figure 2:**
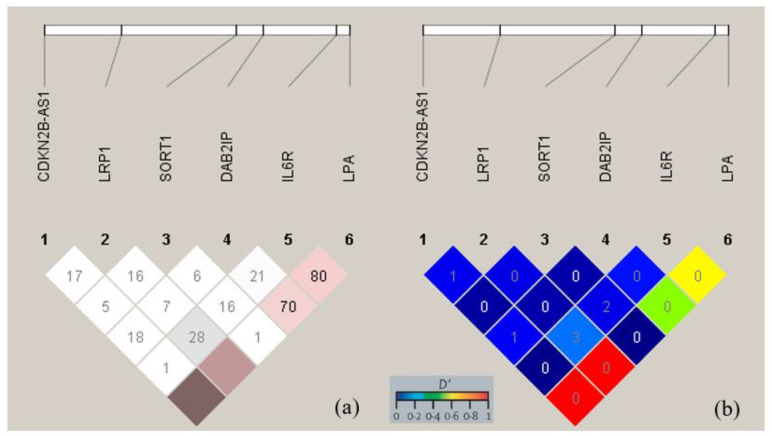
Linkage Disequilibrium (LD) blocks for all SNPs. (**a**) Number in the small square indicates the percentage of the *D’* regarding two SNPs that crossed one another, with the color scheme being the alternate of *D’*/*LOD* (log of likelihood odds ratio) according to Haploview 4.2. White color indicates low *D’*–low *LOD* or low *D’*–high *LOD* (shades of grey indicate higher *D’*); shades of pink to red color indicate high *D’*–low *LOD* (darker pink to red or brown-red indicates higher *D’*); black indicates high *D’*–high *LOD*. Absence of number inside the small square indicates *D’* = 100%. (**b**) Number in the small square indicates the *r^2^* value in percentage, with color scheme of GOLD Heatmap. Shade color from yellowish to red indicates higher *D’*. See Supplementary Table S3 for detailed *D’* and *r^2^* values.

Linkage disequilibrium (LD) describes the nonrandom association of alleles at two or more loci (Figure 2 and Supplementary Table S4). The SNPs *LPA* rs3798220[C] to *DAB2IP* rs7025486[A], to *IL6R* rs2228145[C], to *CDKN2BAS* rs10757278[G], and to *LRP1* rs1466535[T] had *D’* values of 0.709, 0.804, 1.00, and 1.00, respectively. Expressed as the correlation coefficient *r^2^*, the two highest *r^2^* values occurred for *IL6R* rs2228145[C] to *LRP1* rs1466535[T] and for *IL6R* rs2228145[C] to *SORT1* rs599839[G] and reached 3.9% and 2.2%, respectively.

## 4. Discussion

The present study shows that the single nucleotide polymorphisms (SNPs) of the genes *CDKN2BAS* rs10757278, *LPA* rs3798220, *SORT1* rs599839, *DAB2IP* rs7025486, and *IL6R* rs2228145 are associated with the development of abdominal aortic aneurysms (AAA) in a study population. To our knowledge, this is the first study to investigate polymorphisms in the selected SNP panel and to assess the Linkage Disequilibrium (LD) among them.

The SNPs in the processes of apoptosis and inflammation (*CDKN2BAS* rs10757278 +501A>G and *IL6R* rs2228145 +501A>C) and cholesterol metabolism (*LPA* rs3798220 +501T>C and *SORT1* rs599839 +813A>G) represent risk factors for the development of AAA, while the SNP *DAB2IP* rs7025486 +501G>A has a protective effect. The SNP *LRP1* rs1466535 +504C>T is not associated with AAA in the investigated population.

Polymorphisms of AAA have been investigated in different genome-wide association studies producing strong evidence that various SNPs are associated with AAA development [9,10]. Nevertheless, the pathway of these genetic polymorphisms in the development of AAA remains unclear.

The apoptotic process is critical in a physiological way and induced by tumor-suppressing genes [12,13]. *DAB2IP* plays a role in cell growth inhibition and correlates as a tumor-suppressing gene with the apoptotic process of the *CDKN2BAS* mechanism pathway. Histological images of the enlarged aortic wall have shown this apoptotic process [14,15]. The inactivation of tumor-suppressor genes could lead to an increased level of interleukin-6 receptor (IL6R) as demonstrated by Öner et al. [16,17]. Furthermore, IL6 as an inflammation mediator plays a role in the inflammatory process involved in the development of AAA [16,17]. Besides the tumor-suppressing genes, apoptosis, and inflammation theories, the development of AAA has a strong association with hypercholesterolemia [18]. Genetic polymorphisms of *LRP1* (rs1466535), *LPA* (rs3798220), and *SORT1* (rs599839) play a role in cholesterol metabolism [19,20,21]. This supports the theory that the development of AAA is associated with cholesterol metabolism in humans, although the exact pathway remains unclear.

### 4.1. Role of VSMC Apoptosis in Aneurysmal Formation–Potential Involvement of DAB2IP rs7025486[A], SORT1 rs599839[G], and CDKN2BAS rs10757278[G]

In aneurysmal formation, the apoptotic process is apparently due to vascular smooth muscle cell (VSMC) apoptosis which shows an over-expression of p53 in the aneurysmal aortic wall [22]. Both *DAB2IP* and *CDKN2BAS* use the p53 signaling pathway in the apoptotic process [23,24,25]. With reference to the histological images and the p53 signaling in VSMC, this might be a pathway via which *DAB2IP* and *CDKN2BAS* are involved in the development of AAA.

Recent studies demonstrated that *DAB2IP* rs7025486[A] and *SORT1* rs599839[G] showed an association with AAA expansion rate [3]. In the present study, the allele frequencies of *DAB2IP* rs7025486[A] and *SORT1* rs599839[G] were significantly higher in the AAA group than in the control group. Furthermore, the *DAB2IP* rs7025486[A] haplotypes and mutation occurrences displayed significant differences. Our findings are in line with another GWAS, which also found significant data for the [A] allele in *DAB2IP* rs7025486 [26]. *DAB2IP* is located on 9q33.1-q33.3 and acts as an apoptosis signal-regulating kinase 1-interacting protein. This GTP-ase-activating protein plays a role in mediating TNF-induced cell apoptosis and in cell cycle checkpoint regulation. The latter has an inhibitory effect on vascular smooth muscle cell (VSMC) proliferation via the pathway of JAK-STAT and on endothelial cell migration and angiogenesis [3]. The miR-182/SORT1 axis regulates vascular smooth muscle cell calcification in vitro and in vivo [27]. The present study also confirms that *DAB2IP* rs7025486[A] and *SORT1* rs599839[G] are associated with a large diameter of the aneurysm sac.

Additionally, *CDKN2BAS* rs10757278 [G] showed significant differences in allele frequencies, haplotypes, and mutation occurrences and these findings match well with the meta-analysis results of 9p21 (*CDKN2BAS*) A/G and G/G [9,28]. The LD blocks for *CDKN2BAS* rs10757278[G] also had a strong D’ with *LPA* rs3798220[C] (D’ = 100%). This SNP is also significantly associated with metabolic syndrome (MetS) and hypercholesterolemia [29]. p53 signaling is involved in cholesterol metabolism via a process known as the ‘Hippo Pathway’ [30,31].

### 4.2. Involvement of Hypercholesterolemia in Aneurysmal Formation–Potential Roles of SORT1 rs599839 [G], LRP1 rs1466535 [T], and LPA rs3798220 [C]

*SORT1*, besides its function in VSMC calcification, and the genes *LRP1* and *LPA* have a common pathway in cholesterol metabolism [19,25]. Lu et al. also reported that hypercholesterolemia was associated with the development of AAA [32]. A systematic review by Bradley et al. reported a significant association between the genetic polymorphism of *SORT1* rs599839[G] and AAA [9]. The present results also revealed significant differences in this SNP in the [G] allele between the AAA and control groups (*p* = 0.035) and as a risk factor for AAA (*p* = 0.025) in obesity, but not in the haplotype nor in the mutation occurrence (*p* = 0.203 and 0.100). Of note, the *SORT1* rs599839[G] allele was 2.714 times more likely to occur in the AAA group with a minor allele frequency (MAF) of 2.3%.

In our study, the genetic polymorphism of *LRP1* rs1466535 [T] showed no significant differences in allele frequencies, haplotypes, and mutation occurrences but was associated with a family history of AAA (*p* = 0.005) and a large aortic diameter. In the present study, the SNP *LPA* rs3798220[C] showed significant differences in allele frequencies and in the location of AAA but not in the haplotypes nor the mutation occurrence. The present data support the theory that the SNPs *LPA* rs3798220[C] and *SORT1* rs599839[G] but not *LRP1* rs1466535 are associated with the development of AAA.

It has been reported that *LPA* rs3798220 plays a role in cholesterol metabolism, in which the JAK-STAT signaling pathway is crucial [20,33]. It can be assumed that *CDKN2BAS* rs10757278 and LPA rs3798220 stimulate the development of AAA via p53 signaling and the cholesterol pathway. Moreover, the coincidence in strong LD shows that these two SNPs are more closely linked.

Clarke et al. reported that *LPA* rs3798220 accounted for 36% of the low-density lipoprotein variations in systemic atherosclerosis [34]. Due to its correlation with this vascular disease, *LPA* is also implicated in coronary artery disease and PAD, as well as AAA [35,36]. Although the present study did not imply significant results for the *LPA* rs3798220[G] variant in haplotypes and mutation occurrences, this gene contributed in the linkage disequilibrium to a higher D’ value than the other genetic polymorphisms. The consequence of this is that the presence of *LPA* rs3798220 could interfere with the presence of the other SNPs, such as *CDKN2BAS* rs10757278, *LRP1* rs1466535, *IL6R* rs2228145, and *DAB2IP* rs7025486.

### 4.3. Role of Inflammatory Mediators in Aneurysmal Formation–Potential Involvement of IL6R rs2228145[C] and LPA rs3798220[C]

The circulating level of IL6 as an inflammatory mediator was shown to correlate with the presence of AAA [37]. Previous studies showed that the SNP *IL6R* rs2228145[C] had significant differences in haplotypes AC and CC as demonstrated in the downstream effect of IL6R (STAT3) expression [16,25,37,38]. The present study showed significant differences in the incidence of these haplotypes of *IL6R* rs2228145 in the AAA group compared to the control group. It can be assumed that this association is closely related to the inflammation process in the apoptosis pathway in AAA development. Furthermore, according to the Linkage Disequilibrium blocks, this SNP had the highest value of r2 in correspondence with *LRP1* rs1466535 and *SORT1* rs599839.

### 4.4. Limitations of This Study

It was not possible to achieve homogeneity of the “sex and age” of the AAA and the control groups. In the control group, *DAB2IP* rs7025486 and *LPA* rs3798220 were in accordance with the Hardy–Weinberg Disequilibrium, instead of the Hardy–Weinberg Equilibrium.

## 5. Conclusions

Five SNPs’ variation in the selected panel, i.e., *DAB2IP* rs7025486[A], *CDKN2BAS* rs10757278[G], *IL6R* rs2228145[C], *LPA* rs3798220[C], and *SORT1* rs599839[G], are associated with the development of AAA disease. The SNP *LRP1* rs1466535[T], however, is not associated with AAA disease but is associated with a large aortic diameter in AAA. *LPA* rs3798220 is linked predominantly to the other investigated SNPs. This study could be used to inform the genetic screening of AAA patients and their families.

## Figures and Tables

**Table 1:** Sample Characteristics Defined by Group.

Parameter	AAAs, *n* = 148 *n* (%)	Controls, *n* = 50 *n* (%)	*p*-Value
Sex			<0.001
Female	19 (12.8)	27 (54.0)	
Male	129 (87.2)	23 (46.0)	
Age, years	74.8 ± 8.3	49.5 ± 13.2	<0.001
19–35	0 (0)	10 (20.0)	
36–65	22 (14.9)	36 (72.0)	
>65	126 (85.1)	4 (8.0)	
Body Mass Index, kg/m^2^	27.6 ± 4.6	26.3 ± 5.7	0.097
Body height in cm	176.5 ± 7.8	177.5 ± 9.6	0.462
Body weight in kg	86.1 ± 15.0	83.5 ± 23.0	0.358
Hypertension	61 (41.2)	10 (20)	0.07
Systolic in mmHg	133.1 ± 20.0	122.6 ± 11.9	0.001
Diastolic in mmHg	75.9 ± 11.7	73.0 ± 10.6	0.12
MAP	94.9 ± 12.6	89.5 ± 9.7	0.177
Dyslipidemia	66 (44.6)	5 (10.0)	<0.001
Smoking history	95 (64.2)	11 (22)	<0.001
Smoking in pack years	34.9 ± 14.9	20.7 ± 13.7	0.003
PAD	25 (16.9)	6 (12.0)	0.411
Relatives with AAA history	27 (18.2)	3 (6.0)	0.139
Parent–child relationship *	15 (10.1)	1 (2.0)	
Sibling relationship	3 (2.0)	1 (2.0)	
Twin	5 (3.4)	0 (0)	
Others	4 (2.7)	1 (2.0)	
Sex of relatives diagnosed with AAA			0.539
Male–male or female–female	19 (12.8)	2 (4.0)	
Male–female or female–male	7 (4.7)	1 (2.0)	
Both sexes to male/female	1 (0.7)	0 (0)	
Younger than sample	7 (4.7)	2 (4.0)	
Older than sample	20 (13.5)	1 (2.0)	

* paternal relationship (*n* = 9/198, 4.5%) and maternal relationship (*n* = 7/198, 3.5%) in all samples (patients and controls). One sample in the control group was a paternal relationship. Abbreviations: MAP (mean arterial pressure); AAA (abdominal aortic aneurysm); PAD (peripheral arterial disease).

**Table 2:** Genotype Mutation Risk for the Occurrence of AAA with Different Risk Factors.

Variable	*DAB2IP* rs7025486		*LRP1* rs1466535	
	OR [95% CI]	*p*-Value	OR [95% CI]	*p*-Value
Age, years	1.353 [0.679–2.698]	0.389	1.026 [0.534–1.970]	0.938
Male sex	1.874 [0.809–4.341]	0.139	0.873 [0.424–1.796]	0.711
Smoking history	1.661 [0.865–3.193]	0.126	1.002 [0.539–1.863]	0.995
Family history of AAA	2.146 [0.896–5.141]	0.081	3.275 [1.390–7.717]	0.005
First-degree relatives	2.300 [0.869–6.087]	0.087	1.985 [0.753–5.230]	0.159
Obesity	1.229 [0.586–2.581]	0.585	0.665 [0.304–1.454]	0.304
Hypertension	3.295 [1.704–6.374]	<0.001	1.365 [0.723–2.578]	0.337
Dyslipidemia	0.967 [0.497–1.882]	0.922	0.795 [0.413–1.533]	0.494
PAD	1.218 [0.520–2.850]	0.650	1.253 [0.548–2.861]	0.593
Statins	0.967 [0.497–1.882]	0.922	0.795 [0.413–1.533]	0.494
Aspirin	0.813 [0.415–1.589]	0.544	0.596 [0.305–1.166]	0.129
Clopidogrel	3.133 [0.963–10.200]	0.048	1.891 [0.574–6.227]	0.288
Warfarin	1.020 [0.379–2.745]	0.969	1.751 [0.711–4.313]	0.219
**Variable**	***CDKN2BAS* rs10757278**		***IL6R* rs2228145**	
	**OR [95% CI]**	***p*-** **Value**	**OR [95% CI]**	***p*-** **Value**
Age, years	1.344 [0.733–2.467]	0.339	0.496 [0.228–1.078]	0.073
Male sex	1.327 [0.668–2.639]	0.419	0.482 [0.211–1.100]	0.079
Smoking history	1.258 [0.710–2.230]	0.431	0.490 [0.224–1.075]	0.072
Family history of AAA	1.213 [0.520–2.829]	0.654	0.432 [0.096–1.933]	0.260
First-degree relatives	1.401 [0.542–3.621]	0.484	0.609 [0.133–2.777]	0.517
Obesity	0.611 [0.301–1.241]	0.171	0.788 [0.301–2.058]	0.626
Hypertension	1.063 [0.588–1.922]	0.839	0.981 [0.440–2.185]	0.962
Dyslipidemia	1.276 [0.707–2.302]	0.419	0.981 [0.440–2.185]	0.962
PAD	1.509 [0.698–3.260]	0.293	0.768 [0.249–2.373]	0.646
Statins	1.276 [0.707–2.302]	0.419	0.981 [0.440–2.185]	0.962
Aspirin	2.031 [1.126–3.665]	0.018	0.931 [0.419–2.073]	0.862
Clopidogrel	3.239 [0.941–11.151]	0.051	0.473 [0.059–3.799]	0.471
Warfarin	1.182 [0.491–2.844]	0.709	0.788 [0.219–2.830]	0.714
**Variable**	***LPA* rs3798220**		***SORT1* rs599839**	
	**OR [95% CI]**	***p*-** **Value**	**OR [95% CI]**	***p*-** **Value**
Age, years	0.298 [0.069–1.285]	0.087	1.381 [0.620–3.075]	0.428
Male sex	0.488 [0.112–2.123]	0.329	1.572 [0.609–4.059]	0.347
Smoking history	0.863 [0.210–3.551]	0.838	1.847 [0.862–3.960]	0.111
Family history of AAA	0.988 [0.116–8.386]	0.991	1.568 [0.576–4.266]	0.375
First-degree relatives	1.365 [0.159–11.729]	0.561	2.387 [0.839–6.794]	0.095
Obesity	2.114 [0.485–9.212]	0.385	2.419 [1.101–5.314]	0.025
Hypertension	0.245 [0.030–2.032]	0.263	1.654 [0.789–3.466]	0.180
Dyslipidemia	0.585 [0.115–2.976]	0.714	1.906 [0.911–3.991]	0.084
PAD	1.050 [1.015–1.087]	0.362	0.650 [0.212–1.992]	0.610
Statins	0.585 [0.115–2.976]	0.513	1.906 [0.911–3.991]	0.084
Aspirin	0.234 [0.028–1.942]	0.145	1.175 [0.556–2.483]	0.672
Clopidogrel	1.045 [1.014–1.077]	0.463	0.927 [0.194–4.431]	0.925
Warfarin	1.048 [1.014–1.082]	0.600	0.978 [0.311–3.076]	0.970

**Table 3:** Allele Frequencies of Genetic Polymorphism.

			AAAs’ Allele *n* = 296		Controls’ Allele *n* = 100		OR	95% CI	*p*-Value
		Allele	n	%	n	%			
*DAB2IP* rs7025486 + 501G>A	Male	G	212	71.6	40	40.0			
		A	46	15.5	6	6.0	0.691	0.277–1.727	0.427
	Female	G	32	10.8	51	51.0			
		A	6	2.1	3	3.0	0.314	0.073–1.344	0.154
	Total	G	244	82.4	91	91.0			
		A	52	17.6	9	9.0	0.464	0.220–0.980	0.040
*LRP1* rs1466535 + 504C>T	Male	C	219	74.0	36	36.0			
		T	39	13.2	10	10.0	0.641	0.294–1.397	0.260
	Female	C	28	9.5	47	47.0			
		T	10	3.4	7	7.0	2.398	0.820–7.014	0.104
	Total	C	247	83.4	83	83.0			
		T	49	16.6	17	17.0	0.969	0.529–1.774	0.918
*CDKN2BAS* rs10757278 + 501A>G	Male	A	184	62.2	38	38.0			
		G	74	25.0	8	8.0	1.910	0.851–4.289	0.112
	Female	A	28	9.5	45	45.0			
		G	10	3.4	9	9.0	1.786	0.646–4.935	0.260
	Total	A	212	71.6	83	83.0			
		G	84	28.4	17	17.0	1.935	1.083–3.454	0.024
*IL6R* rs2228145 + 501A>C	Male	A	240	81.1	41	41.0			
		C	18	6.1	5	5.0	0.615	0.216–1.748	0.358
	Female	A	34	11.5	47	47.0			
		C	4	1.4	7	7.0	0.790	0.214–2.914	0.723
	Total	A	274	92.6	88	88.0			
		C	22	7.4	12	12.0	0.589	0.280–1.238	0.159
*LPA* rs3798220 + 501T>C	Male	T	254	85.8	44	44.0			
		C	4	1.4	2	2.0	2.886	0.513–16.24	0.226
	Female	T	38	12.8	51	51.0			
		C	0	0.0	3	3.0	0.944	0.885–1.008	0.265
	Total	T	292	98.6	95	95.0			
		C	4	1.4	5	5.0	3.842	1.011–14.60	0.049
*SORT1* rs599839 + 813A>G	Male	A	226	76.4	44	44.0			
		G	32	10.8	2	2.0	3.115	0.720–13.47	0.132
	Female	A	33	11.1	51	51.0			
		G	5	1.7	3	3.0	2.576	0.577–11.51	0.268
	Total	A	259	87.5	95	95.0			
		G	37	12.5	5	5.0	2.714	1.036–7.110	0.035

**Table 4:** Haplotypes in Genetic Polymorphism.

		Haplotype *n* (%)							MAF * (%)
		Major Homozygote		Heterozygote		Minor Homozygote		*p*-Value	
		AAAs	Controls	AAAs	Controls	AAAs	Controls		
*DAB2IP* rs7025486 + 501G>A	Male	90 (69.8)	19 (82.6)	32 (24.8)	2 (8.7)	7 (5.4)	2 (8.7)		
	Female	14 (73.7)	24 (88.9)	4 (21.1)	3 (11.1)	1 (5.2)	0 (0)		
	Total	104 (70.3)	43 (86.0)	36 (24.3)	5 (10.0)	8 (5.4)	2 (4.0)	0.037	25.5
*LRP1* rs1466535 + 504C>T	Male	95 (73.6)	15 (65.2)	29 (22.5)	6 (26.1)	5 (3.9)	2 (8,7)		
	Female	12 (63.2)	20 (74.1)	4 (21.1)	7 (25.9)	3 (15.7)	0 (0)		
	Total	107 (72.3)	35 (70.0)	33 (22.3)	13 (26.0)	8 (5.4)	2 (4.0)	0.835	16.7
*CDKN2BAS* rs10757278 + 501A>G	Male	72 (55.8)	17 (73.9)	40 (31.0)	4 (17.4)	17 (13.2)	2 (8.7)		
	Female	10 (52.6)	19 (70.4)	8 (42.1)	7 (25.9)	1 (5.3)	1 (3.7)		
	Total	82 (55.4)	36 (72.0)	48 (32.4)	11 (22.0)	18 (12.2)	3 (6.0)	0.037	10.6
*IL6R* rs2228145 + 501A>C	Male	114 (88.4)	18 (78.3)	12 (9.3)	5 (21.7)	3 (2.3)	0 (0)		
	Female	15 (78.9)	20 (74.1)	4 (21.1)	7 (25.9)	0 (0)	0 (0)		
	Total	129 (87.2)	38 (76.0)	16 (10.8)	12 (24.0)	3 (2.0)	0 (0)	0.046	15.4
*LPA* rs3798220 + 501T>C	Male	125 (96.9)	22 (95.7)	4 (3.1)	0 (0)	0 (0)	1 (4.3)		
	Female	19 (100.0)	24 (88.9)	0 (0)	3 (11.1)	0 (0)	0 (0)		
	Total	144 (97.3)	46 (92.0)	4 (2.7)	3 (6.0)	0 (0)	1 (2.0)	0.146	8.6
*SORT1* rs599839 + 813A>G	Male	102 (79.0)	21 (91.3)	22 (17.1)	2 (8.7)	5 (3.9)	0 (0)		
	Female	16 (84.2)	24 (88.9)	1 (5.3)	3 (11.1)	2 (10.5)	0 (0)		
	Total	118 (79.7)	45 (90.0)	23 (15.5)	5 (10.0)	7 (4.8)	0 (0)	0.203	2.3

* MAF, minor allele frequency.

**Table 5:** Mutation Occurrences in each Genetic Polymorphism.

		Mutation (n, %)				
		Occurred		Not Occurred		*p*-Value
		AAAs	Controls	AAAs	Controls	
*DAB2IP* rs7025486 + 501G>A	Male	39 (30.2)	4 (17.4)	90 (69.8)	19 (82.6)	0.314
	Female	5 (26.3)	3 (11.1)	14 (73.7)	24 (88.9)	0.246
	Total	44 (29.7)	7 (14.0)	104 (70.4)	43 (86.0)	0.028
*LRP1* rs1466535 + 504C>T	Male	34 (26.4)	8 (34.8)	95 (73.6)	15 (65.2)	0.405
	Female	7 (36.8)	7 (25.9)	12 (63.2)	20 (74.1)	0.428
	Total	41 (27.7)	15 (30.0)	107 (72.3)	35 (70.0)	0.755
*CDKN2BAS* rs10757278 + 501A>G	Male	57 (44.2)	6 (26.1)	72 (55.8)	17 (73.9)	0.105
	Female	8 (42.1)	8 (29.6)	11 (57.9)	19 (70.4)	0.382
	Total	65 (43.9)	14 (28.0)	83 (56.1)	36 (72.0)	0.047
*IL6R* rs2228145 + 501A>C	Male	15 (11.6)	5 (21.7)	114 (88.4)	18 (78.3)	0.186
	Female	4 (21.1)	7 (25.9)	15 (78.9)	20 (74.1)	>0.995
	Total	19 (12.8)	12 (24.0)	129 (87.2)	38 (76.0)	0.073
*LPA* rs3798220 + 501T>C	Male	4 (3.1)	1 (4.3)	125 (96.9)	22 (95.7)	0.565
	Female	0 (0)	3 (11.1)	19 (100.0)	24 (88.9)	0.257
	Total	4 (2.7)	4 (8.0)	144 (97.3)	46 (92.0)	0.113
*SORT1* rs599839 + 813A>G	Male	27 (20.9)	2 (8.7)	102 (79.1)	21 (91.3)	0.250
	Female	3 (15.8)	3 (11.1)	16 (84.2)	24 (88.9)	0.680
	Total	30 (20.3)	5 (10.0)	118 (79.7)	45 (90.0)	0.100

**Table 6:** Genotype Risk in Size of Aortic Diameter, AAA Morphology, and AAA Renally Referenced Location.

SNPs	Aneurysmal Sac Size				Morphology of AAA			
	Small (<50 mm) n (%)	Large (≥ 50 mm) *n* (%)	OR [95% CI]	*p*-Value *	Fusiform *n* (%)	Saccular *n* (%)	OR [95% CI]	*p*-Value *
	*n* = 45	*n* = 103			*n* = 111	*n* = 19		
*DAB2IP* rs7025486								
GG	34 (75.6)	70 (68.0)	1.457	<0.001	77 (69.4)	16 (84.2)	2.35	<0.001
GA + AA	11 (24.4)	33 (32.0)	[0.66–3.23]		34 (30.6)	3 (15.8)	[0.64–8.62]	
*LRP1* rs1466535								
CC	35 (77.8)	72 (69.9)	1.507	<0.001	81 (73.0)	15 (78.9)	1.389	<0.001
CT + TT	10 (22.2)	31 (30.1)	[0.66–3.42]		30 (27.0)	4 (21.1)	[0.43–4.52]	
*CDKN2BAS* rs10757278								
AA	24 (53.3)	59 (57.3)	0.852	<0.001	62 (55.9)	10 (52.6)	0.878	<0.001
AG + GG	21 (46.7)	44 (42.7)	[0.42–1.72]		49 (44.1)	9 (47.4)	[0.33–2.33]	
*IL6R* rs2228145								
AA	41 (91.1)	88 (85.4)	1.747	<0.001	96 (86.5)	16 (84.2)	0.833	<0.001
AC + CC	4 (8.9)	15 (14.6)	[0.55–5.59]		15 (13.5)	3 (15.8)	[0.22–3.21]	
*LPA* rs3798220								
TT	45 (100)	99 (96.1)	0.961	0.180	107 (96.4)	19 (100)	0.964	0.401
TC + CC	0 (0)	4 (3.9)	[0.92–1.00]		4 (3.6)	0 (0)	[0.93–1.00]	
*SORT1* rs599839								
AA	35 (77.8)	83 (80.6)	0.843	<0.001	86 (77.5)	16 (84.2)	1.550	<0.001
AG + GG	10 (22.2)	20 (19.4)	[0.36–1.98]		25 (22.5)	3 (15.8)	[0.42–5.75]	
**SNPs**	**Location of AAA**							
	**Supra/Juxtarenal n (%)**	**Infrarenal n (%)**	**OR** **[95% CI]**	*p*-Value *				
	***n* = 53 **	*n* = 95						
*DAB2IP* rs7025486								
GG	37 (69.8)	67 (70.5)	1.035	0.317				
GA + AA	16 (30.2)	28 (29.5)	[0.50–2.15]					
*LRP1* rs1466535								
CC	34 (64.2)	73 (76.8)	1.854	0.138				
CT + TT	19 (35.8)	22 (23.2)	[0.89–3.87]					
*CDKN2BAS* rs10757278								
AA	28 (52.8)	55 (57.9)	1.228	0.118				
AG + GG	25 (47.2)	40 (42.1)	[0.62–2.41]					
*IL6R* rs2228145								
AA	47 (88.7)	82 (86.3)	0.805	<0.001				
AC + CC	6 (11.3)	13 (13.7)	[0.29–2.26]					
*LPA* rs3798220								
TT	51 (96.2)	93 (97.9)	1.824	<0.001				
TC + CC	2 (3.8)	2 (2.1)	[0.25–13.33]					
*SORT1* rs599839								
AA	43 (81.1)	75 (78.9)	0.872	0.005				
AG + GG	10 (18.9)	20 (21.1)	[0.37–2.03]					

* *p*-value indicates the Chi-square test between groups.

## Data Availability

Data available on request from the authors.

## Supplementary Materials

The following supporting information can be downloaded at: https://www.mdpi.com/article/10.3390/medicina59101844/s1, (Figure S1: PCR Results in Electrophoresis Gel for all SNPs; Table S1: List of Primers; Table S2: AAA Patients’ Characteristics Defined by Sex; Table S3: Hardy–Weinberg (HW) Calculation; Table S4: Linkage Disequilibrium for Genetic Polymorphism).
