# Supplementary material for: Association of Genetic Polymorphisms with Abdominal Aortic Aneurysm in the Processes of Apoptosis, Inflammation, and Cholesterol Metabolism
Source: Medicina (Kaunas). 2023 Oct 17;59(10):1844. doi: 10.3390/medicina59101844 (PMC10608078; doi:10.3390/medicina59101844)
Supplement: Supplementary file 1 [file medicina-59-01844-s001.zip › medicina-2595647-supplementary.pdf]

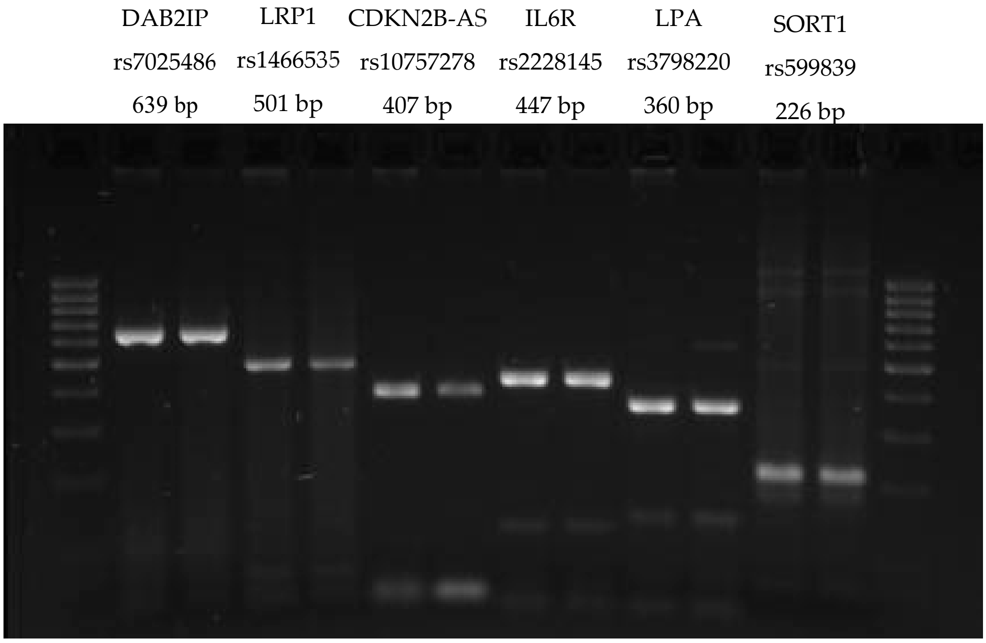

Figure S1. PCR Results in Electrophoresis Gel for all SNPs. This figure represented the PCR products of two samples with their base pairs (bp) length. The first bandwidth of each genetic polymorphism was the first sample (patient), and the second bandwidth was the second sample (control). The DNA ladder (the outer left and right bandwidth) is shown in a 100 bp distance, starting with 1000 bp.

Table S1. List of Primers.

Gene Name	Forward and Reverse Primers
DAB2IP rs7025486 +501G>A	Sense 5′ - CATCCCTACCCTGAGAGGCT - 3′<br>Antisense 5′ –GCCGGCCTCCTTCAAAATTC - 3′(639 bp)
LRP1 rs1466535 +504C>T	Sense 5′- TCCCATGGACCCCAGTGCCTCAACAT - 3′<br>Antisense 5′ – CTGCAACCTGGGAGCTATGG - 3′ (501 bp)
CDKN2BAS rs10757278 +501A>G	Sense 5′- CTTCTAAACTAACAAACAGCCAATTTG - 3′<br>Antisense 5′ – AGCTGAGACGACTTCTGGCCCT - 3′ (407 bp)
IL6R rs2228145 +501A>C	Sense 5′- AACCCTGAGCTTGAGGTGTC - 3′<br>Antisense 5′ – TTCAGAATGGGCAAAGGAAAGC - 3′ (447 bp)
LPA rs3798220 +501T>C	Sense 5′ – AGGGCTGGGGTTGAAGATTG – 3′<br>Antisense 5′ – AGAGGATACCTGAAGGGGCT – 3′ (360 bp)
SORT1 rs599839 +813A>G	Sense 5′ – GGCTGAGAAAGGAGAATTGCTTGAACCC – 3′<br>Antisense 5′ – TAATAGGCCAGTTTGTGATAAGCTTAC – 3′ (226 bp)

Table S2. AAA Patients' Characteristics Defined by Gender.

Parameter	Female, n=19<br>n (%)	Male, n=129<br>n (%)	p-value
Pre-op aortic diameter (mm)	54.4 ± 11,9	57.9 ± 11.8	.228
<50*	6 (31.6)	39 (30.2)	
≥50	13 (68.4)	90 (69.8)	
Type of AAA			.005
Fusiform	11 (57.9)	100 (77.5)	
Saccular	1 (5.3)	**18 (14.0)**	
TAAA	7 (36.8)	**11 (8.5)**	
Renal association			**.001**
Suprarenal	8 (42.1)	**13 (10.1)**	
Juxtarenal	1 (5.3)	**31 (24.0)**	
Infrarenal	10 (52.6)	**85 (65.9)**	
Diagnosed as AAA in years	4.8 ±2.7	**5.9 ± 6.3**	**.473**
Treatment for AAA			**.545**
Endovascular repair	18 (94.7)	**104 (80.6)**	
Open surgery repair	1 (5.3)	**22 (17.1)**	
Re-intervention	0 (0)	**3 (2.3)**	
Lower extremity claudication history	2 (10.5)	**12 (9.3)**	**>.995**
Abdominal pain history	1 (5.3)	**5 (3.9)**	**.568**
Medication history			
Statin	6 (31.6)	**60 (46.5)**	**.222**
Acetylsalicylic acid	12 (63.2)	**61 (47.3)**	**.196**
Clopidogrel	1 (5.3)	**11 (8.5)**	**>.995**
Warfarin	3 (15.8)	**20 (15.5)**	**>.995**

*operation by indication

Table S3. Hardy-Weinberg (HW) Calculation.

	HW			
	AAAs		*Controls*	
	χ2	*p*	χ2	*p*
DAB2IP rs7025486 +501G>A	3.796	.051	7.585	.006
LRP1 rs1466535 +504C>T	5.509	.019	0.309	.578
CDKN2BAS rs10757278 +501A>G	6.048	.014	2.429	.119
IL6R rs2228145 +501A>C	6.799	.009	0.929	.335
LPA rs3798220 +501T>C	0.028	.868	6.787	.009
SORT1 rs599839 +813A>G	12.410	.000	0.138	.709

*Hardy-Weinberg Equilibrium (HWE) occurs if p-value ≥.05, and Hardy-Weinberg Disequilibrium (HWD) occurs if p-value <.05. HWE declares a steady-state of allele frequency in the normal population from generation to generation.

Table S4. Linkage Disequilibrium for Genetic Polymorphism.

	***DAB2IP* rs7025486 +501G>A**		***LRP1* rs1466535 +504C>T**		***CDKN2BAS* rs10757278 +501A>G**		***IL6R* rs2228145 +501A>C**		***LPA* rs3798220 +501T>C**		***SORT1* rs599839 +813A>G**	
	**D'**	**r^2**	**D'**	**r^2**	**D'**	**r^2**	**D'**	**r^2**	**D'**	**r^2**	**D'**	**r^2**
***DAB2IP* rs7025486 +501G>A**			0.070	0.004	0.186	0.018	0.212	0.001	0.709	0.002	0.067	0.003
***LRP1* rs1466535 +504C>T**	0.070	0.004			0.177	0.018	0.288	0.039	1.000	0.005	0.162	0.001
***CDKN2BAS* rs10757278 +501A>G**	0.186	0.018	0.177	0.018			0.016	0.000	1.000	0.008	0.056	0.001
***IL6R* rs2228145 +501A>C**	0.212	0.001	0.288	0.039	0.016	0.000			0.804	0.001	0.165	0.022
***LPA* rs3798220 +501T>C**	0.709	0.002	1.000	0.005	1.000	0.008	0.804	0.001			0.013	0.000
***SORT1* rs599839 +813A>G**	0.067	0.003	0.162	0.001	0.056	0.001	0.165	0.022	0.013	0.000		
